# Prospective validation of the BOADICEA multifactorial breast cancer risk prediction model in a large prospective cohort study

**DOI:** 10.1136/jmg-2022-108806

**Published:** 2022-09-26

**Authors:** Xin Yang, Mikael Eriksson, Kamila Czene, Andrew Lee, Goska Leslie, Michael Lush, Jean Wang, Joe Dennis, Leila Dorling, Sara Carvalho, Nasim Mavaddat, Jacques Simard, Marjanka K Schmidt, Douglas F Easton, Per Hall, Antonis C Antoniou

**Affiliations:** 1 Centre for Cancer Genetic Epidemiology, Department of Public Health and Primary Care, University of Cambridge, Strangeways Research Laboratory, Cambridge, UK; 2 Department of Medical Epidemiology and Biostatistics, Karolinska Institutet, Stockholm, Sweden; 3 Department of Molecular Medicine, Université Laval and CHU de Québec-Université Laval Research Center, Quebec City, Quebec, Canada; 4 Department of Clinical Genetics, Leiden University Medical Center, Leiden, The Netherlands; 5 Devision of Molecular Pathology, The Netherlands Cancer Institute, Antoni van Leeuwenhoek Hospital, Amsterdam, The Netherlands; 6 Centre for Cancer Genetic Epidemiology, Department of Oncology, University of Cambridge, Cambridge, UK; 7 Department of Oncology, Södersjukhuset, Stockholm, Sweden

**Keywords:** genetic counseling, public health, women's health

## Abstract

**Background:**

The multifactorial Breast and Ovarian Analysis of Disease Incidence and Carrier Estimation Algorithm (BOADICEA) breast cancer risk prediction model has been recently extended to consider all established breast cancer risk factors. We assessed the clinical validity of the model in a large independent prospective cohort.

**Methods:**

We validated BOADICEA (V.6) in the Swedish KARolinska Mammography Project for Risk Prediction of Breast Cancer (KARMA) cohort including 66 415 women of European ancestry (median age 54 years, IQR 45–63; 816 incident breast cancers) without previous cancer diagnosis. We calculated 5-year risks on the basis of questionnaire-based risk factors, pedigree-structured first-degree family history, mammographic density (BI-RADS), a validated breast cancer polygenic risk score (PRS) based on 313-SNPs, and pathogenic variant status in 8 breast cancer susceptibility genes: *BRCA1*, *BRCA2*, *PALB2*, *CHEK2*, *ATM*, *RAD51C*, *RAD51D* and *BARD1*. Calibration was assessed by comparing observed and expected risks in deciles of predicted risk and the calibration slope. The discriminatory ability was assessed using the area under the curve (AUC).

**Results:**

Among the individual model components, the PRS contributed most to breast cancer risk stratification. BOADICEA was well calibrated in predicting the risks for low-risk and high-risk women when all, or subsets of risk factors are included in the risk prediction. Discrimination was maximised when all risk factors are considered (AUC=0.70, 95% CI: 0.66 to 0.73; expected-to-observed ratio=0.88, 95% CI: 0.75 to 1.04; calibration slope=0.97, 95% CI: 0.95 to 0.99). The full multifactorial model classified 3.6% women as high risk (5-year risk ≥3%) and 11.1% as very low risk (5-year risk <0.33%).

**Conclusion:**

The multifactorial BOADICEA model provides valid breast cancer risk predictions and a basis for personalised decision-making on disease prevention and screening.

**What is already known on this topic**

- No study has assessed the clinical validity of the full multifactorial Breast and Ovarian Analysis of Disease Incidence and Carrier Estimation Algorithm (BOADICEA) model for predicting future breast cancer risks.

**What this study adds**

- This is the first study to validate the comprehensive BOADICEA model based on the joint effects of family history, questionnaire-based risk factors, mammographic density, polygenic risk score and rare pathogenic variants in all eight established breast cancer susceptibility genes.
- The model is well calibrated overall and predicts risks accurately in different categories of predicted risk.
- It discriminates well between affected and unaffected women and can result in clinically meaningful levels of breast cancer risk stratification.

**How this study might affect research, practice or policy**

- BOADICEA is freely available via the CanRisk tool (www.canrisk.org) and has been incorporated in several clinical management guidelines in the UK, North America and other countries.
- BOADICEA can be used by healthcare professionals in personalising risk assessment to facilitate shared decision-making with patients on lifestyle changes, prevention or screening options for managing breast cancer risk.
- It can be used to identify high-risk women who may benefit most from enhanced screening or other preventive or risk-reducing treatments, and also to identify low-risk women who are unlikely to benefit from such interventions, which are also associated with adverse effects.
- The results are based mainly on data from women of European ancestry. Further model customisation and validation is needed before applying the model to women from other ancestry groups.

## Introduction

Breast cancer (BC) is the most common cancer diagnosed among women worldwide and is associated with significant mortality.^1^ Screening and prevention options are available, including mammography, MRI, chemoprevention and risk-reducing surgery, but these are costly and may be associated with overdiagnosis, overtreatment or adverse effects.^2–4^ BC risk prediction models have the potential of improving individualised BC risk assessment and population risk stratification.^5^ They can be used for identifying women at high risk who are most likely to benefit from such interventions.

The Breast and Ovarian Analysis of Disease Incidence and Carrier Estimation Algorithm (BOADICEA) model as implemented in the CanRisk tool (www.canrisk.org) is currently used by clinicians to estimate the future risk of developing BC.^6–11^ The latest V.6 models BC risk using seven types of risk factors^12 13^: (1) pathogenic variants (PVs) in eight high-risk or moderate-risk BC susceptibility genes *BRCA1*, *BRCA2*, *PALB2*, *CHEK2*, *ATM*, *RAD51C*, *RAD51D* and *BARD1*; (2) a validated polygenic risk score (PRS), using 313 common BC genetic susceptibility variants^14^; (3) detailed cancer family history (FH), including age at cancer diagnosis for affected relatives and age at last follow-up or death for unaffected relatives; (4) a residual polygenic component accounting for familial aggregation not explained by the above observed genetic effects; (5) nine questionnaire-based lifestyle, hormonal and reproductive risk factors (QRFs); (6) mammographic density (MD) measured using the BI-RADS breast composition categorisation^15^ and (7) demographic factors including age, year of birth and country. This is the first comprehensive BC risk prediction model considering all established BC risk factors, MD, PRS and PVs in all eight established BC susceptibility genes and is hypothesised to improve risk stratification compared with older versions of BOADICEA that considered only FH and *BRCA1/2* PV information.^16 17^

Previous prospective validation studies have been either small or were based on women ascertained in high-risk settings; assessed older versions of BOADICEA, based only on FH and *BRCA1/2* PV information or used only a subset of the risk factors considered in the model.^18–21^ None assessed the full multifactorial model. Here, for the first time, we evaluate the performance of the full BOADICEA model^12 13^ in an independent population-based prospective cohort of >60 000 women.

## Methods

### Study participants

The KARolinska Mammography Project for Risk Prediction of Breast Cancer (KARMA) cohort is a large prospective population-based cohort of women who were invited to the Swedish national mammography screening programme between January 2011 and March 2013.^22^ In the Swedish national screening programme, women are invited from age 40 to 74 years. Approximately 3% of the women are recalled following a screening mammogram for further investigation due to a suspicious finding. Additionally, approximately 0.2% of the women have a mammogram between scheduled screens due to symptoms or clinical referral. The present study included regularly screened women and symptomatic women who had mammograms performed on screening machines at study baseline at four hospitals in Sweden. The women were followed for BC events until 2017 with a mean/median follow-up of 5.7/6.0 years).^22^ All participants completed a baseline questionnaire, which included anthropometric/lifestyle/hormonal and reproductive factors. Full-field digital processed mammograms of right and left breasts were collected at baseline to measure MD using the area-based STRATUS software, which estimated the MD on digital processed mammograms using a machine learning method.^23^ The percentage of MD was then calculated as the ratio of the dense area to the total area, which were categorised into the four computer-assessed BI-RADS categories by fixed thresholds (<2%, 2-8%, 8-49% and >49%).^15 23 24^ The resulting age-specific BI-RADS distributions were in line with data from the general population^25^ and used in BOADICEA, although there is a somewhat higher proportion of women in category ‘A’ in particular among women aged 50 years or older (online supplemental table S1). Self-reported FH data were coded as pedigrees, including information on ages at BC diagnosis or last observation for first-degree relatives. Personal cancer diagnosis and death were obtained through linkages with high-quality healthcare registers, which captured 98.5% of all incident cancers within 12 months of diagnosis.^22 26^ For this analysis, the last linkage was performed in June 2017. Each participant’s follow-up was censored at date of BC (invasive or in situ), bilateral prophylactic mastectomy, last linkage date, death, baseline plus 5 years or age 80 years, whichever occurred first. We restricted our analyses to women with no previous cancer (any) or risk-reducing mastectomy history who had information on FH, QRFs and MD.

10.1136/jmg-2022-108806.supp1 Supplementary data

### Genetic data

Genotyping and gene-panel sequencing data were available for a subset of the cohort based on previous genotyping experiments.^27 28^ Genotyping for the 313-SNP PRS^14^ was performed on the majority of the BC incident cases available and randomly selected unaffected women from the entire cohort that reflected the distribution in the entire KARMA cohort. A subset of the participants with PRS was sequenced for a 34-gene panel through the Breast Cancer Risk after Diagnositic GEne Sequencing (BRIDGES) study^27^ using the same sampling approach. For this subcohort, PV carrier status was determined in the *BRCA1*, *BRCA2*, *PALB2*, *CHEK2*, *ATM*, *RAD51C*, *RAD51D* and *BARD1* genes. The risk factor distributions for all QRFs (including any missing categories) were virtually the same in the entire KARMA cohort and the two subcohorts with genetic data (online supplemental table S2).

### Risk prediction

We predicted 5-year BC risks using BOADICEA V.6,^12^ with Swedish age-specific and calendar period-specific population incidences for invasive BC. To exclude potentially prevalent patients with undiagnosed BC at study recruitment, we predicted the 5-year BC risks starting from the age at baseline plus 1 year. Women were considered as affected only if they developed invasive BC within this 5-year risk prediction interval. For unaffected women with follow-up time shorter than 5 years, the BC risks were predicted to the censored age. Since BOADICEA allows for missing questionnaire-based risk factor information,^12^ risk predictions were carried out for all women irrespective of individual missing QRFs. Predictions involving genetic data were carried out only in the subcohorts of women with the genetic data available (see "Statistical analysis").

### Statistical analysis

Model calibration was assessed by the ratio of the expected (E) to the observed (O) BC risk in the dataset.^29 30^ We also assessed the agreement between predicted and observed risk for each individual using the calibration slope, which was calculated by fitting a logistic regression where the dependent variable was the observed outcome (affected/unaffected) and the independent variable was the log-odds of the predicted risk. The calibration slope assesses whether the predicted risks are too extreme or conversely too moderate, especially at the high-risk and low-risk tails and is expected to be equal to 1 if the model is perfectly calibrated. The observed and expected risks were also compared in decile categories of predicted risks or by menopausal status at recruitment. Discrimination was assessed by the area under the receiver operating characteristic curve (AUC) and Harrell’s concordance (C) index.^31–33^ Bootstrap (100 replications) was used to obtain CIs for Harrell’s C-index estimates.^31 32^

We assessed the contribution of individual and combinations of risk factors in predicting BC risk. All participants had information on age, year of birth, QRFs, FH, MD and were from Sweden. Of those, 15 502 participants had also information on PRS and 5693 had PRS and gene-panel sequencing data. When assessing the model performance in the subcohorts, to avoid potential biases we considered the explicit sampling scheme by using an inverse probability weighted approach,^21 29 34^ which reflects the probability of participants being included in the subcohort. The sample inclusion probabilities were computed by fitting a logistic regression model in the full dataset in which the outcome (inclusion or not) was dependent on the age at baseline, follow-up duration, incident BC status and the interaction between BC status, age at baseline and the follow-up duration.^34^ The weights were then the inverse of the fitted inclusion probabilities for each individual. To allow for a direct comparison of the models with or without genetic factors, model comparison were performed in the subcohorts with genetic information.

To evaluate the potential improvements in risk classification when adding QRFs, MD, PRS and gene-panel sequencing data, to FH (previous version of BOADICEA), we calculated the proportion of all participants and incident BCs reclassified as high risk. Women with 5-year BC risks above 3% were considered as high risk, in line with the risk threshold recommended for risk-reducing treatments by the US Preventive Services Task Force.^35^

All analyses were performed in R (V.3.6.3)^36^ and the R package ‘iCare’ was used to calculate the E/O and AUC.^29^ Sensitivities and specificities were calculated using R package ‘epiR’.^37^ All statistical tests were two-sided.

## Results

**Figure 1:**
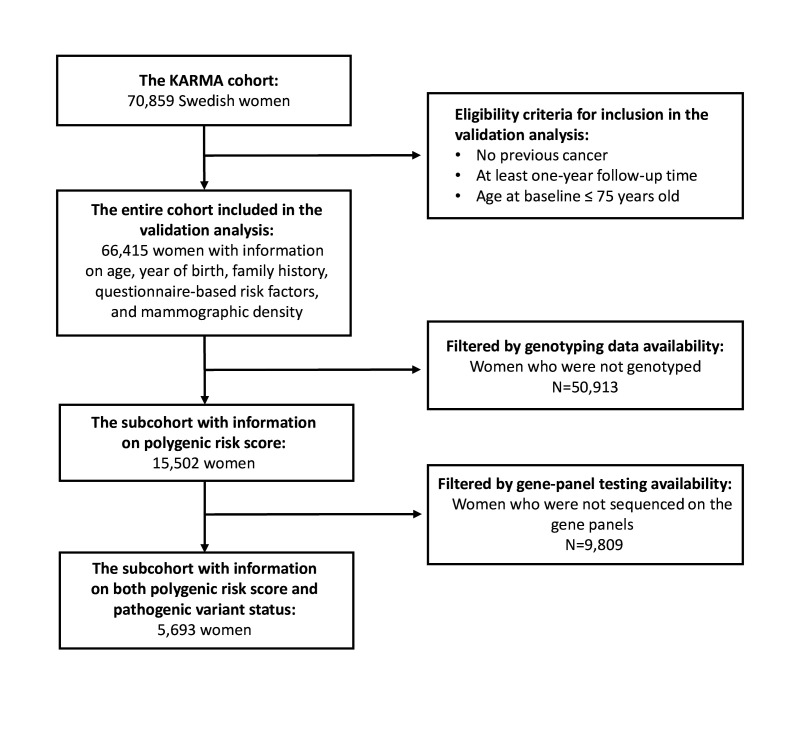
Consolidated Standards of Reporting Trials diagram summarising the KARolinska Mammography Project for Risk Prediction of Breast Cancer (KARMA) cohort data.

A total of 66 415 participants (aged 25–74 years) were eligible for inclusion in the analysis with information available on FH, QRFs and MD (figure 1, online supplemental table S2). Among these, 816 developed BC within the 5-year risk prediction horizon (tumour characteristics in online supplemental table S3); 90.1% were of self-reported European ancestry (online supplemental table S2). Of the eligible participants, 15 502 women (23.3%) had information on the 313-SNP PRS (676 affected) and 5693 women (8.6%) had PRS and PV status in *BRCA1*, *BRCA2*, *PALB2*, *CHEK2*, *ATM, RAD51C*, *RAD51D* and *BARD1*, of whom 280 were affected (figure 1, online supplemental table S2). A detailed summary of the genetic and epidemiological characteristics of participants at baseline are shown in online supplemental table S2).

### Model discrimination and calibration

Using the entire cohort, FH, QRFs or MD individually resulted in similar estimated AUCs and Harrell’s C-indexes of 0.62 (table 1, online supplemental figure S1). The AUC was maximised when FH, QRFs and MD were considered jointly (AUC=0.64, 95% CI: 0.62 to 0.66). For this model, the overall ratio of expected to observed number of cases (E/O) was 0.92 (95% CI: 0.86 to 0.99) with a slight underestimation of risk in the middle deciles of predicted risk when MD was included (online supplemental figure S2). However, the model was well-calibrated in the bottom and top deciles of predicted risk. The calibration slope was 0.98 (95% CI: 0.96 to 1.00, table 1).

**Figure 2:**
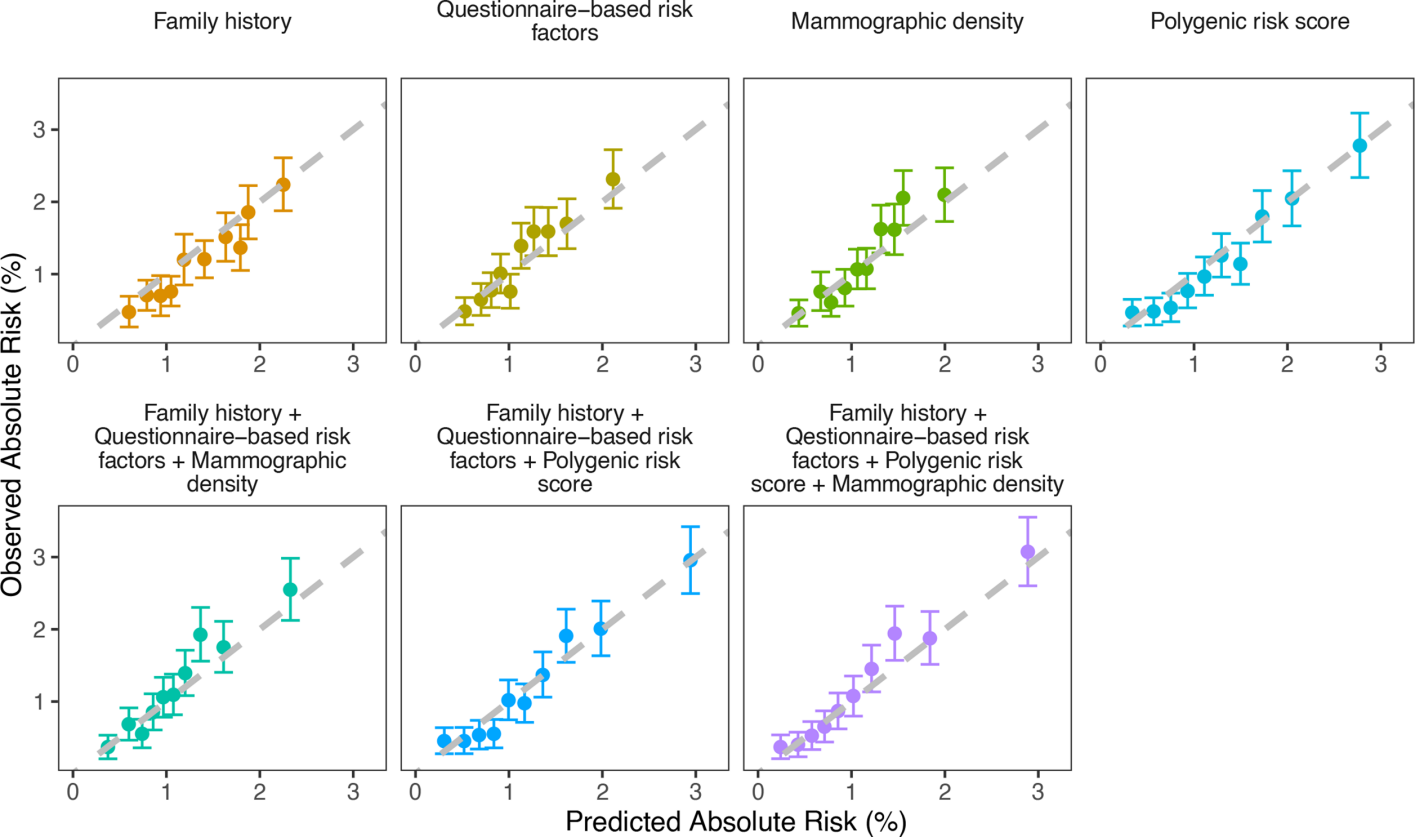
Observed and predicted 5-year breast cancer risks using the subcohort of participants with polygenic risk score information (n=15 502) under different risk factor combinations. Women were grouped into deciles of predicted risks. Each dot represents the mean observed and predicted risk in the decile and the vertical segments represent 95% CIs. The dashed line is the diagonal line with slope equal to 1 (corresponding to expected to observed number of cases ratio of 1 for each decile). When the CI crosses the diagonal, the decile-predicted risk is not significantly different from the observed risk. When a dot and the associated CI fall above the diagonal, there is a suggestion for underprediction of risk; when a dot and associated CI fall below the diagonal, there is a suggestion for overprediction of risk.

**Table 1:** Calibration and discrimination of 5-year predicted breast cancer risks under the BOADICEA model using different risk factor combinations

Model	AUC (95% CI)	Harrell’s C-index (95% CI)	E/O (95% CI)	Calibration slope (95% CI)
Entire cohort with information on FH, QRFs and MD (n=66 415; n.BCs=816)				
FH	0.62 (0.60 to 0.64)	0.62 (0.60 to 0.64)	1.12 (1.05 to 1.20)	1.03 (1.01 to 1.05)
QRFs	0.62 (0.60 to 0.64)	0.62 (0.60 to 0.64)	0.95 (0.89 to 1.02)	0.99 (0.97 to 1.01)
MD	0.62 (0.60 to 0.64)	0.62 (0.60 to 0.64)	0.94 (0.88 to 1.01)	0.99 (0.97 to 1.01)
FH+QRFs	0.63 (0.62 to 0.65)	0.63 (0.61 to 0.64)	1.02 (0.95 to 1.09)	1.01 (0.99 to 1.02)
FH+QRFs+MD	0.64 (0.62 to 0.66)	0.64 (0.61 to 0.66)	0.92 (0.86 to 0.99)	0.98 (0.96 to 1.00)
Subcohort with information on FH, QRFs, MD and PRS (n=15 502; n.BCs=676)				
FH	0.61 (0.59 to 0.64)	0.63 (0.60 to 0.65)	1.12 (1.04 to 1.21)	1.03 (1.01 to 1.05)
QRFs	0.63 (0.61 to 0.65)	0.63 (0.61 to 0.65)	0.94 (0.87 to 1.01)	0.99 (0.97 to 1.01)
MD	0.63 (0.61 to 0.65)	0.64 (0.62 to 0.66)	0.93 (0.87 to 1.01)	0.99 (0.97 to 1.00)
PRS	0.67 (0.64 to 0.69)	0.67 (0.65 to 0.69)	1.06 (0.99 to 1.15)	1.02 (1.00 to 1.03)
FH+QRFs+MD	0.65 (0.63 to 0.67)	0.65 (0.63 to 0.67)	0.91 (0.84 to 0.98)	0.98 (0.96 to 1.00)
FH+QRFs+PRS	0.68 (0.66 to 0.70)	0.68 (0.66 to 0.70)	1.01 (0.94 to 1.09)	1.00 (0.98 to 1.02)
FH+QRFs+PRS+MD	0.69 (0.67 to 0.71)	0.69 (0.67 to 0.71)	0.92 (0.85 to 0.99)	0.98 (0.96 to 1.00)
Subcohort with information on FH, QRFs, MD, PRS and PV status (n=5693; n.BCs=280)				
FH+QRFs+PRS+MD	0.69 (0.64 to 0.73)	0.70 (0.67 to 0.73)	0.88 (0.74 to 1.04)	0.97 (0.95 to 0.99)
FH+QRFs+PRS+MD+PV	0.70 (0.66 to 0.73)	0.71 (0.68 to 0.74)	0.88 (0.75 to 1.04)	0.97 (0.95 to 0.99)

BC, breast cancer; BOADICEA, Breast and Ovarian Analysis of Disease Incidence and Carrier Estimation Algorithm; C-index, concordance index; E, expected number of BCs in the 5-year period; FH, family history; MD, mammographic density in BI-RADS; n.BCs, number of patients with incident breast cancer; O, observed number BCs; PRS, polygenic risk score; PV, pathogenic variants in *BRCA1, BRCA2, PALB2, CHECK2, ATM, RAD51C, RAD51D* and *BARD1*; QRFs, questionnaire-based risk factors.

The estimated calibration and discrimination statistics for models that considered FH, QRFs or MD individually or jointly were similar in the weighted subcohort of women with PRS information and the full cohort (table 1), indicating no evidence of bias due to sampling. The PRS provided the widest distribution of predicted risks among the individual model components (online supplemental figure S3), and discriminated best between patients with incident BC and unaffected women (AUC=0.67, 95% CI: 0.64 to 0.69, table 1). The calibration slope for this model was 1.02 (95% CI: 1.00 to 1.03) and the overall E/O 1.06 (95% CI: 0.99 to 1.15). When the PRS was combined with FH and QRFs, both discrimination and calibration were improved (AUC=0.68, 95% CI: 0.66 to 0.70; calibration slope=1.00, 95% CI: 0.98 to 1.02; E/O=1.01, 95% CI: 0.94 to 1.09; figure 2, table 1). The addition of MD into the model further improved the discrimination (AUC=0.69, 95% CI: 0.67 to 0.71, table 1) but resulted in some risk underprediction (E/O=0.92, 95% CI: 0.85 to 0.99) mainly in the eighth decile of predicted risks, although there was no evidence for systematic underprediction (calibration slope=0.98, 95% CI: 0.96 to 1.00, figure 2).

**Figure 3:**
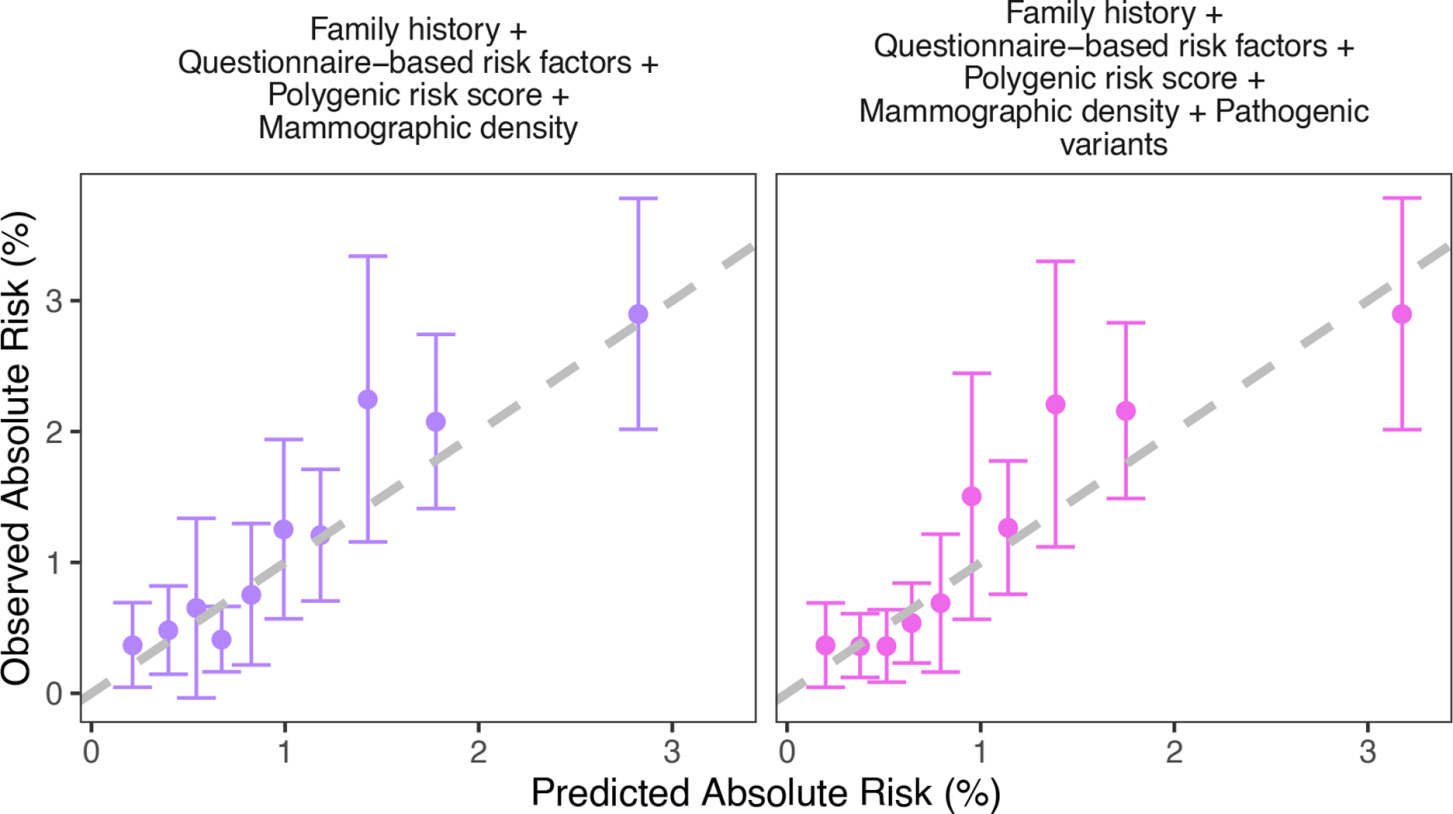
Observed and predicted 5-year breast cancer risks using the subcohort of participants with polygenic risk score and pathogenic variant status information (n=5 693) under different models. Women were grouped into deciles of predicted risks.

Using the weighted subcohort of women with both PRS and PV information, the full model including FH, QRFs, MD, PRS and PVs predicted the widest BC risk distribution (online supplemental figure S4) and maximised model discrimination (AUC=0.70, 95% CI: 0.66 to 0.73, table 1). The calibration slope was 0.97 (95% CI: 0.95 to 0.99 and the overall E/O 0.88 (95% CI: 0.75 to 1.04, table 1, figure 3). The possible underprediction was primarily driven by the inclusion of MD (for the model without MD: E/O=1.00, 95% CI: 0.84 to 1.17, calibration slope=1.00, 95% CI: 0.98 to 1.02). However, the model was well calibrated in the bottom and high deciles of predicted risk (figure 3).

**Figure 4:**
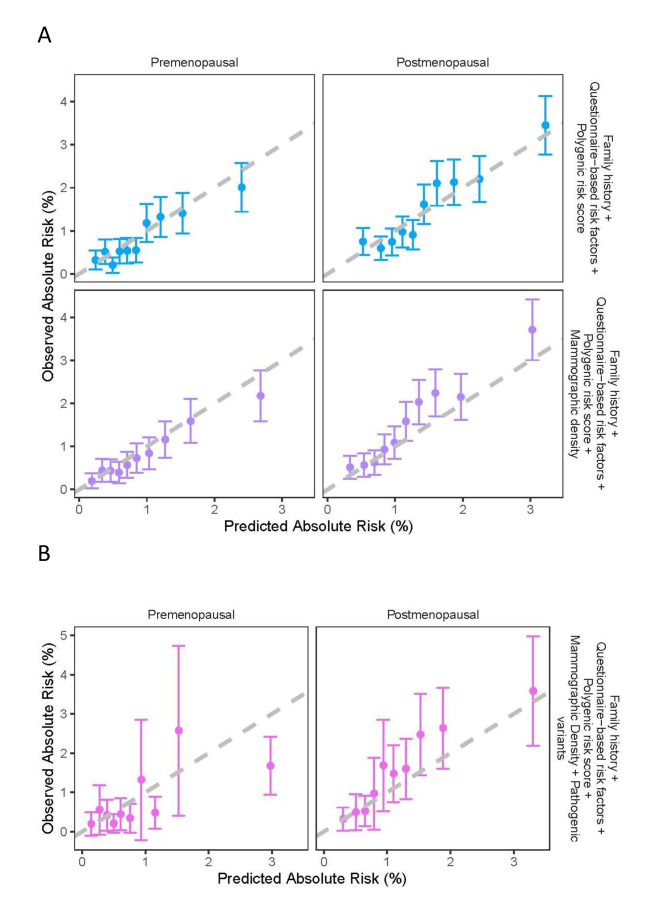
Observed and predicted 5-year breast cancer (BC) by menopausal status, using (A) the subcohort of participants with polygenic risk score (PRS) information under the model considering family history, questionnaire-based risk factors and PRS and the model considering family history, questionnaire-based risk factors, PRS and mammographic density in BI-RADS and (B) the subcohort of participants with PRS and pathogenic variant status under the model considering family history, questionnaire-based risk factors, PRS, mammographic density in BI-RADS and pathogenic variant status in the eight major BC susceptibility genes: *BRCA1*, *BRCA2*, *PALB2*, *CHECK2*, *ATM*, *RAD51C*, *RAD51D* and *BARD1*. Women were grouped into deciles of predicted risks.

### Model performance by menopausal status

Among women with PRS data, there were 5714 premenopausal (212 BCs) and 9788 postmenopausal women (464 BCs). When considering FH, QRFs and PRS, the model was well calibrated in both premenopausal (calibration slope=1.02, 95% CI: 0.99 to 1.05) and postmenopausal women (calibration slope=0.99, 95% CI: 0.97 to 1.02). The AUC for premenopausal women was 0.68 (95% CI: 0.64 to 0.71) compared with 0.66 (95% CI: 0.63 to 0.68) in postmenopausal women; table 2, figure 4A). When MD was added, the AUC was increased for both premenopausal (AUC=0.69, 95% CI: 0.65 to 0.72) and postmenopausal women (AUC=0.67, 95% CI: 0.64 to 0.69, table 2). However, there was some underprediction of risk among postmenopausal women, for the middle seventh-eighth deciles of predicted risk (figure 4). The overall calibration slopes in premenopausal and postmenopausal women were 1.03 (95% CI: 1.00 to 1.06) and 0.95 (95% CI: 0.93 to 0.97), respectively. Among women with PRS and PV information, there were 1293 premenopausal (77 incident cancers) and 4400 postmenopausal women (203 incident cancers). When considering the full multifactorial model including PVs, the AUC in premenopausal women was 0.69 (95% CI: 0.62 to 0.76) and in postmenopausal women 0.68 (95% CI: 0.64 to 0.73). There was an overall underprediction of risk among postmenopausal women under the full model (E/O=0.78, 95% CI: 0.65 to 0.93; calibration slope=0.94, 95% CI: 0.92 to 0.96, table 2, figure 4B), but there was no significant difference between observed and predicted risk for any of the risk deciles. The full model without MD was well calibrated overall (E/O=0.94, 95% CI: 0.79 to 1.13; calibration slope=0.99, 95% CI: 0.96 to 1.01).

**Table 2:** Calibration and discrimination of 5-year predicted breast cancer risks by menopausal status

Menopausal status	n. unaffected	n. BCs	Model	AUC (95% CI)	Harrell’s C-index (95% CI)	E/O (95% CI)	Calibration slope (95% CI)
Subcohort with information on FH, QRFs, MD and PRS (n=15 502; n.BCs=676)							
Premenopausal	5502	212	FH+QRFs+PRS	0.68 (0.64 to 0.71)	0.67 (0.62 to 0.70)	1.10 (0.96 to 1.26)	1.02 (0.99 to 1.05)
			FH+QRFs+PRS+MD	0.69 (0.65 to 0.72)	0.68 (0.65 to 0.71)	1.14 (1.00 to 1.31)	1.03 (1.00 to 1.06)
Postmenopausal	9324	464	FH+QRFs+PRS	0.66 (0.63 to 0.68)	0.65 (0.63 to 0.68)	0.97 (0.89 to 1.06)	0.99 (0.97 to 1.02)
			FH+QRFs+PRS+MD	0.67 (0.64 to 0.69)	0.67 (0.64 to 0.69)	0.81 (0.74 to 0.89)	0.95 (0.93 to 0.97)
Subcohort with information on FH, QRFs, MD, PRS and PV status (n=5693; n.BCs=280)							
Premenopausal	1216	77	FH+QRFs+PRS+PV	0.70 (0.62 to 0.79)	0.68 (0.62 to 0.74)	1.11 (0.77 to 1.60)	1.02 (0.99 to 1.06)
			FH+QRFs+PRS+PV+MD	0.69 (0.62 to 0.76)	0.69 (0.62 to 0.76)	1.13 (0.79 to 1.62)	1.02 (0.99 to 1.05)
Postmenopausal	4197	203	FH+QRFs+PRS+PV	0.68 (0.63 to 0.73)	0.69 (0.66 to 0.72)	0.94 (0.79 to 1.13)	0.99 (0.96 to 1.01)
			FH+QRFs+PRS+PV+MD	0.68 (0.64 to 0.73)	0.69 (0.66 to 0.73)	0.78 (0.65 to 0.93)	0.94 (0.92 to 0.96)

BC, breast cancer; C-index, concordance index; E, expected number of BCs in the 5-year period; FH, family history; MD, mammographic density in BI-RADS; n.BCs, number of patients with incident breast cancer; n.unaffected, number of unaffected women; O, observed number BCs; PRS, polygenic risk score; PV, pathogenic variants in *BRCA1, BRCA2, PALB2, CHECK2, ATM, RAD51C, RAD51D* and *BARD1*; QRFs, questionnaire-based risk factors.

### Risk classification, sensitivity/specificity

The model considering FH alone classified 0.8% women as high risk (5-year risk ≥3%), including 1.7% of the patients with incident BC. The addition of QRFs, PRS and MD reclassified 2.8% women from the low-risk (risk <3%) to the high-risk (risk ≥3%) group and 0.5% in the opposite direction (online supplemental table S4). Under this model, the sensitivity was 69%, 38% and 9% at the ≥1.1%, ≥1.67% and ≥3% risk thresholds (online supplemental table S5); and was 37%, 53% and 65% when considering the top 20%, 30% and 40% of the predicted risks distribution, respectively (online supplemental table S6).

The addition of PV information reclassified 1.0% women from the low-risk to the high-risk group and 0.3% from the high-risk to the low-risk group (online supplemental table S7). Under this model, 3.6% women were classified as high risk, including 8.9% of the patients with incident BC during the 5-year risk prediction horizon.

## Discussion

This is the first study to validate the comprehensive BOADICEA model^12^ in predicting future BC risks using the joint effects of FH, QRFs, MD, PRS and rare PVs. The results show that the model is well calibrated both overall and in predicting the risks for low-risk and high-risk women. The model is well calibrated when all, or subsets of risk factors are included. However, the discrimination is maximised when all risk factors are considered with an AUC of 0.70 (table 1). When assessing each risk factor individually, the PRS contributed most to the BC risk stratification.

Previous validation studies^18 19^ evaluated older versions of BOADICEA^16^ and have shown good discriminatory ability and calibration in women with BC FH.^19^ Detailed multigenerational pedigree data were included in those studies, as opposed to only first-degree relatives in this study which may be less informative. Two smaller population-based studies have assessed BOADICEA V.5 using only FH, QRFs and PRS.^20 21^ These studies suggested that FH, QRFs and PRS jointly predicted risks well in high-risk and low-risk groups and had AUCs of 0.62 for predicting 10-year^20^ and 0.65–0.70 for predicting 5-year risks.^21^ As different populations were used, the reported AUCs, which depend on the age distribution and other characteristics of the populations, are not directly comparable across studies. In the present study, the results show that compared with previous versions of BOADICEA that considered only FH information, the full multifactorial model improves discrimination (AUC increases from 0.61 to 0.70, table 1) and identifies 3.6% women as high risk (5-year BC risk ≥3%) compared with 0.8% when only FH is considered.

The inclusion of the 313-SNP PRS led to the greatest improvement in discriminatory ability, followed by MD, QRFs and first-degree FH. These patterns are in line with theoretical expectations under the BOADICEA model^12^ and results from other studies^34 38–40^ and further support the inclusion of PRS in routine multifactorial risk assessment in clinical practice, for improving risk stratification.^41^ Here, we used the 313-SNP PRS.^14^ However, BOADICEA allows for alternative PRS to be used as long as the population distribution characteristics of the PRS are known.^12^

This study also suggests that BOADICEA is well calibrated overall and in deciles of predicted risks for all risk factors individually except for MD where there was some underprediction in the middle deciles, mainly among postmenopausal women (figure 4). This may represent some misspecification of the BI-RADS classification in the KARMA cohort. The BI-RADS classification was derived from continuous MD measurements generated using STRATUS^23^ based on specific ‘%MD’ category thresholds. It does not correspond exactly to the visually assessed BI-RADS categories. For example, the underprediction in the seventh–eighth deciles of predicted risks using the model considering FH, QRFs, PRS and MD in postmenopausal women may be due to some misclassification between BI-RADS levels B and C when converting ‘%MD’ to BI-RADS (online supplemental figure S6). Nevertheless, BOADICEA was well calibrated in the highest and lowest BC risk groups for both premenopausal and postmenopausal women. Even for the visually assessed BI-RADS, previous studies showed that there is substantial variability in BI-RADS categorisation between radiologists.^42^ Since %MD has a stronger association with BC than categorised BI-RADS,^43^ we would expect an improvement in BC risk prediction when incorporating continuous MD as risk factor in the model in the future.

Provided a model is calibrated in predicting risks in different risk categories, its clinical utility for prevention or early detection will depend on its ability to risk stratify the population by identifying groups of individuals in the population with sufficient differences in absolute risk to warrant the adoption of certain interventions. Therefore, the clinical utility of a model depends both on the discriminatory ability as measured by the AUC and the population risk of the disease.^38 44^ For a common disease, such as BC here, the results demonstrate that even modest increases in AUC can lead to a substantial increase in overall levels of risk stratification which are clinically meaningful. Taking the 5-year BC risk of 3% as a high-risk threshold, recommended for the risk-reducing treatments,^8 34^ under the full model, 3.6% women were classified as high risk, identifying 8.9% of the patients with incident BC during the 5-year risk prediction horizon. Alternatively, taking the 5-year BC risk of 1.67% as a ‘moderate-risk’ threshold, recommended for preventive risk reducing treatments in the USA,^4 45^ 16.9% women in the study were classified above this threshold, including 34.0% patients with incident BC. Taking the 5-year BC risk of 0.33% as a very-low-risk threshold (equivalent to a relative risk of 0.3 relative to the population risk at age 50), 88.9% women were above the threshold, identifying 96.6% patients with incident BC. The remaining 11.1% women identified as very-low-risk may opt for less intensive screening. In practice, optimal risk thresholds may need to be determined which consider the balance of benefits and harms from specific screening or early detection or prevention options.^46^

This study has some limitations.^31 32^ Information on the PRS and PV status was available only for subsets of the participants. To maximise power, the subset with genetic information included the majority of incident BC cases and a random sample of unaffected women from the entire cohort. Direct model evaluation using these subcohorts would have been susceptible to bias if the sampling scheme was ignored in the analysis. Here, we used a weighted cohort analytical approach^29 34^ that considers the probability of a genotyped individual being sampled from the entire cohort, which yields unbiased estimates for the model discriminatory ability and calibration. This is demonstrated in the results for the risk prediction models that could be evaluated both in the entire cohort and the weighted subcohorts: models that included QRFs, FH and MD, individually or combined (table 1). The model discriminatory ability and calibration estimates were almost identical in the entire cohort and the two subcohorts indicating no evidence of bias due to sampling.

Previous studies indicated that the PRS and other risk factors modify the BC risks for PV carriers and can lead to significantly different levels of risk stratification specifically for PV carriers.^47–51^ Our results showed that PV carriers had a wider BC risk distribution than non-carriers (online supplemental figure S5). However, we were not able to assess the detailed model performance separately for women with high-penetrance and moderate-penetrance PVs due the small number of PV carriers in the dataset (only 13 carriers developed BCs). Although at population level the increase in AUC when including gene-panel testing information on top of other risk factors and the PRS is modest, the change in personalised risk and risk stratification is large for those carrying PVs. As an example, among the 110 PV carriers, prior to the inclusion of gene-panel testing information, 74 had 5-year risks <1.67%, 30 had risks between 1.67% and 3% and 6 had risks >3%. After the inclusion of the gene-panel testing information, these numbers were 28, 25 and 57, respectively. The corresponding estimated E/O was improved from 0.31 (95% CI: 0.13 to 0.74) when no gene-panel testing information was considered to 0.99 (95% CI: 0.42 to 2.35) after including gene-panel testing information (online supplemental table S8).^15 43 52^ Although the subcohort with gene-panel testing information was also included in the study by Dorling *et al*,^27^ this is unlikely to bias the results here because the penetrance parameters for PVs in most of the genes included in BOADICEA V.6 had been previously obtained from other studies.^12 13 17^ In BOADICEA V.6, only the BC relative risks for *BARD1* and *ATM* variants depend solely on BRIDGES data.^12^ The BC relative risks for *RAD51C* and *RAD51D* PVs used in BOADICEA come from the meta-analysis of the Yang *et al* 53 and Dorling *et al,* 27 where the estimates were virtually identical.

KARMA is based on women attending mammographic screening, so the participants tend to be more highly educated and have a healthier lifestyle and may not be entirely representative of the general population.^54^ Nevertheless, the results will be applicable to similar populations participating in screening programmes, one of the main settings in which risk stratification is used. KARMA included a small number of women who had mammography under 40 years old (0.4% of women), the minimum age entering the Swedish national screening programme. After excluding these women, the results remained similar (online supplemental table S9-S10, figure S7-S8) in terms of calibration and discrimination. Finally, the KARMA study participants were from Sweden, with the majority being of European ancestry. Therefore, further validation studies are required to assess the model validity in other ethnic subgroups and other countries with different BC incidences.

In summary, the comprehensive BOADICEA model is well calibrated and discriminates well between patients with incident BC and unaffected women undergoing mammographic screening. It is therefore a clinically valid model that can be used to identify high-risk individuals who may benefit most from enhanced screening or other preventive risk-reducing treatments. It can also be used to identify low-risk individuals who are unlikely to benefit from prevention options associated with adverse effects. This validated model can be used as the basis for risk-based feasibility or acceptability studies or randomised controlled trials for determining the optimal risk thresholds, the uptake of different interventions and for health economics analyses that evaluate the balance between benefits and harms and associated healthcare costs from introducing multifactorial risk assessment, including the PRS, at different healthcare levels.^7^ BOADICEA is available via the CanRisk webtool www.canrisk.org 6 that can be used by healthcare professionals in personalising risk assessment to facilitate shared decision-making with patients on lifestyle changes, prevention or screening options for managing BC risk.

## Data Availability

Data are available on reasonable request. Data are available under the Findability, Accessibility, Interoperability, Reproducibility (FAIR) principles, where all data necessary to reproduce the aims of the study are available from Karolinska Institutet through the MTA form available at https://karmastudy.org/contact/data-access/
