# Supplementary material for: Prospective validation of the BOADICEA multifactorial breast cancer risk prediction model in a large prospective cohort study
Source: J Med Genet. 2022 Sep 26;59(12):1196–205. doi: 10.1136/jmg-2022-108806 (PMC9691822; doi:10.1136/jmg-2022-108806)
Supplement: Supplementary data [file jmg-2022-108806supp001.pdf]

Supplementary Materials

METHODS

SNP genotyping and gene-panel testing

Blood samples were collected at recruitment and were processed in the Karolinska Institutet high-throughput Biobank[1]. A subset of the samples, including the majority of BCs, were genotyped using either iCOGS[2, 3] or OncoArray[4] in studies organised through the Breast Cancer Association Consortium (BCAC). Here, we used only the prospective arm of the KARMA study. Genotype quality control was performed as previously described[5]. Genotypes for SNPs not presented on the arrays were imputed using the 1000 Genomes Project (phase 3) as the reference panel[5]. The 313-SNP PRS was derived and standardised by the mean (-0.424) and standard deviation (0.603) as described by Mavaddat et al.[6]. A subset of these samples was sequenced for a 34-gene panel through the Breast Cancer Risk after Diagnostic Gene Sequencing (BRIDGES) study. Details of library preparation, sample sequencing, variant calling and quality control have been described previously[7]. Women were considered as PV carriers in *BRCA1*, *BRCA2*, *PALB2*, *CHEK2*, *ATM*, *RAD51C*, *RAD51D*, or *BARD1* if a protein-truncating (frameshift, nonsense or canonical splice site variant) or a known pathogenic missense variant was identified, with the exception of variants identified in the last exon of each gene.

REFERENCES

1. Gabrielson M, Eriksson M, Hammarstrom M, et al. Cohort Profile: The Karolinska Mammography Project for Risk Prediction of Breast Cancer (KARMA). *Int J Epidemiol* 2017;**46**(6):1740-41g doi: 10.1093/ije/dyw357[published Online First: 2017/02/10].
2. Michailidou K, Beesley J, Lindstrom S, et al. Genome-wide association analysis of more than 120,000 individuals identifies 15 new susceptibility loci for breast cancer. *Nat Genet* 2015;**47**(4):373-80 doi: 10.1038/ng.3242[published Online First: 2015/03/10].
3. Michailidou K, Hall P, Gonzalez-Neira A, et al. Large-scale genotyping identifies 41 new loci associated with breast cancer risk. *Nat Genet* 2013;**45**(4):353-61, 61e1-2 doi: 10.1038/ng.2563[published Online First: 2013/03/29].
4. Amos CI, Dennis J, Wang Z, et al. The OncoArray Consortium: A Network for Understanding the Genetic Architecture of Common Cancers. *Cancer Epidemiol Biomarkers Prev* 2017;**26**(1):126-35 doi: 10.1158/1055-9965.EPI-16-0106
5. Genomes Project C, Auton A, Brooks LD, et al. A global reference for human genetic variation. *Nature* 2015;**526**(7571):68-74 doi: 10.1038/nature15393[published Online First: 2015/10/04].

Yang X, *et al. J Med Genet* 2022; 59:1196–1205. doi: 10.1136/jmg-2022-108806

6. Mavaddat N, Michailidou K, Dennis J, et al. Polygenic Risk Scores for Prediction of Breast Cancer and Breast Cancer Subtypes. *Am J Hum Genet* 2019;**104**(1):21-34 doi: 10.1016/j.ajhg.2018.11.002[published Online First: 2018/12/18].
7. Breast Cancer Association C, Dorling L, Carvalho S, et al. Breast Cancer Risk Genes - Association Analysis in More than 113,000 Women. *N Engl J Med* 2021;**384**(5):428-39 doi: 10.1056/NEJMoa1913948[published Online First: 2021/01/21].
8. Tice JA, Cummings SR, Smith-Bindman R, et al. Using clinical factors and mammographic breast density to estimate breast cancer risk: development and validation of a new predictive model. *Ann Intern Med* 2008;**148**(5):337-47 doi: 10.7326/0003-4819-148-5-200803040-00004[published Online First: 2008/03/05].

Table s1 Comparison of distribution of STRATUS dereived BI-RADS categories in the KARMA cohort and previously published study[8].

BI-RADS Category	In KARMA	Tice *et al*.[8]
**Age < 50y**		
A	0.05	0.04
B	0.24	0.35
C	0.50	0.47
D	0.20	0.14
**Age 50-64y**		
A	0.13	0.07
B	0.42	0.47
C	0.38	0.40
D	0.06	0.06
**Age ⩾ 65y**		
A	0.21	0.11
B	0.50	0.54
C	0.26	0.32
D	0.03	0.03

Yang X, *et al. J Med Genet* 2022; 59:1196–1205. doi: 10.1136/jmg-2022-108806

Table s2 A summary of genetic and epidemiological characteristics of participants at baseline in (1) the entire KARMA cohort (2) sub-cohort samples with PRS information and (3) sub-cohort samples with both PRS and pathogenic variant (PV) status in *BRCA1*, *BRCA2*, *PALB2*, *CHECK2*, *ATM*, *RAD51C*, *RAD51D*, and *BARD1*. The incident BC patients were women who developed breast cancer within five-year risk prediction horizon.

	**Full cohort**		**Sub-cohort with PRS**		**Sub-cohort with PRS and PV**	
	**Healthy women**	**Incident BC patients**	**Healthy women**	**Incident BC patients**	**Healthy women**	**Incident BC patients**
**Number of participants, *N***						
	65,599	816	14,826	676	5,413	280
**Follow-up, median (IQR), years**						
	6.0 (5.0-6.0)	3.0 (2.0-4.0)	6.0 (5.0-6.0)	3.0 (2.0-4.0)	6.0 (5.0-6.0)	3.0 (2.0-4.0)
**European ancestry, *N* (%)**						
**Yes**	59,102 (90.1%)	757 (92.8%)	14,264 (96.2%)	633 (93.6%)	5,307 (98.0%)	267 (95.4%)
**No**	1,642 (2.5%)	16 (2.0%)	223 (1.5%)	9 (1.3%)	2 (0.04%)	4 (1.4%)
**Missing**	4,855 (7.4%)	43 (5.2%)	339 (2.3%)	34 (5.1%)	104 (1.9%)	9 (3.2%)
**Age at baseline, *N* (%)**						
**<40**	263 (0.4%)	0 (0%)	75 (0.5%)	0 (0%)	40 (0.7%)	0 (0.0%)
**40–49**	23,618 (36.0%)	177 (21.7%)	4,048 (27.3%)	140 (20.7%)	695 (12.8%)	56 (20.0%)
**50–59**	18,275 (27.9%)	231 (28.3%)	3,951 (26.7%)	196 (29.0%)	1,338 (24.7%)	77 (27.5%)
**≥60**	23,443 (35.7%)	408 (50.0%)	6,752 (45.5%)	340 (50.3%)	3,340 (61.7%)	147 (52.5%)
**Mean (sd), years**	55 (9.8)	58 (9.4)	57 (10.0)	59 (9.3)	61 (9.1)	59 (9.2)
**Age at menarche*, *N* (%)**						
**<11**	6,828 (10.4%)	79 (9.7%)	728 (4.9%)	62 (9.2%)	250 (4.6%)	18 (6.4%)
**[11,12)**	6,251 (9.5%)	69 (8.5%)	1,487 (10.0%)	53 (7.8%)	512 (9.5%)	27 (9.6%)
**[12,13)**	13,085 (20.0%)	164 (20.1%)	3,106 (21.0%)	132 (19.5%)	1,043 (19.3%)	50 (17.9%)
**[13,14)**	16,759 (25.5%)	216 (26.5%)	3,935 (26.5%)	189 (28.0%)	1,403 (25.9%)	77 (27.5%)
**[14,15)**	13,127 (20.0%)	175 (21.4%)	3,210 (21.6%)	148 (21.9%)	1,230 (22.7%)	70 (25.0%)

**[15,16)**	6,625 (10.1%)	74 (9.1%)	1,654 (11.2%)	61 (9.0%)	692 (12.8%)	27 (9.6%)
**≥16**	2,924 (4.5%)	39 (4.8%)	706 (4.8%)	31 (4.6%)	283 (5.2%)	11 (3.9%)
**Menopausal status, *N* (%)**						
**Pre-menopausal**	29,829 (45.5%)	268 (32.8%)	5,502 (37.1%)	212 (31.4%)	1,216 (22.5%)	77 (27.5%)
**Post-menopausal**	35,770 (54.5%)	548 (67.2%)	9,324 (62.9%)	464 (68.6%)	4,197 (77.5%)	203 (72.5%)
**Age at menopause (among post-menopausal women)*, *N* (%)**						
**<40**	721 (2.0%)	12 (2.2%)	170 (1.8%)	9 (1.9%)	75 (1.8%)	5 (2.5%)
**[40,45)**	1,466 (4.1%)	23 (4.2%)	368 (3.9%)	21 (4.5%)	147 (3.5%)	10 (4.9%)
**[45,50)**	4,116 (11.5%)	75 (13.7%)	1,037 (11.1%)	65 (14.0%)	435 (10.4%)	32 (15.8%)
**[50,55)**	7,706 (21.5%)	135 (24.6%)	1,889 (20.3%)	117 (25.2%)	842 (20.1%)	58 (28.6%)
**≥55**	2,640 (7.4%)	57 (10.4%)	742 (8.0%)	49 (10.6%)	355 (8.4%)	27 (13.3%)
**Missing**	19,121(53.4%)	246 (44.9%)	5,118(54.9%)	203 (43.8%)	2,343(55.8%)	71 (35.0%)
**Use of hormonal replacement treatment (among post-menopausal women)*, *N* (%)**						
**Current C**	655 (1.8%)	19 (3.5%)	152 (1.6%)	14 (3.0%)	55 (1.3%)	4 (2.0%)
**Current E**	806 (2.3%)	25 (4.6%)	198 (2.1 %)	18 (3.9%)	84 (2.0%)	5 (2.5%)
**Former**	8,005 (22.4%)	138 (25.2%)	2,197 (23.6%)	116 (25.0%)	1,058 (25.2%)	50 (24.6%)
**Never**	20,657 (57.7%)	283 (51.6%)	5,650 (60.6%)	244 (52.6%)	2,493 (59.4%)	114 (56.1%)
**Missing**	5,647 (15.8%)	83 (15.1%)	1,127 (12.1%)	72 (15.5%)	507 (12.1%)	30 (14.8%)
**Parity*, *N* (%)**						
**0**	7,785 (11.9%)	94 (11.5%)	1,809 (12.2%)	80 (11.8%)	609 (11.2%)	34 (12.1%)
**1**	8,935 (13.6%)	127 (15.6%)	2,164 (14.6%)	108 (16.0%)	788 (14.6%)	54 (19.3%)
**2**	29,380 (44.8%)	377 (46.2%)	7,007 (47.3%)	312 (46.1%)	2,562 (47.3%)	115 (41.1%)
**≥3**	15,202 (23.2%)	170 (20.8%)	3,740 (25.2%)	137 (20.3%)	1,445 (26.7%)	65 (23.2%)
**Missing**	4,297 (6.5%)	48 (5.9%)	106 (0.7%)	39 (5.8%)	9 (0.2%)	12 (4.3%)
**Age at first live birth (among parious women)*, *N* (%)**						
**<20**	3,108 (5.8%)	42 (6.2%)	842 (6.5%)	36 (6.5%)	366 (7.6%)	15 (6.4%)
**[20,25)**	14,915 (27.9%)	183 (27.2%)	3,815 (29.5%)	145 (26.0%)	1,560 (32.5%)	57 (24.3%)
**[25,30)**	18,847 (35.2%)	240 (35.6%)	4,562 (35.3%)	203 (36.4%)	1,783 (37.2%)	83 (35.5%)
**≥30**	16,625 (31.1%)	209 (31.0%)	3,682 (28.5%)	173 (31.1%)	1,081 (22.5%)	79 (33.8%)
**Missing**	22 (0.04%)	0 (0.0%)	10 (0.1%)	0 (0.0%)	5 (0.1%)	0 (0.0%)

Yang X, *et al. J Med Genet* 2022; 59:1196–1205. doi: 10.1136/jmg-2022-108806

**Use of oral contraceptive*, *N* (%)**						
**Ever**	51,882 (79.1%)	632 (77.5%)	12,198 (82.3%)	526 (77.8%)	4,307 (79.6%)	221 (78.9%)
**Never**	8,834 (13.5%)	134 (16.4%)	2,341 (15.8%)	110 (16.3%)	1,013 (18.7%)	46 (16.4%)
**Missing**	4,883 (7.4%)	50 (6.1%)	287 (1.9%)	40 (5.9%)	93 (1.7%)	13 (4.6%)
**Body Mass Index* (kg/m2), *N* (%)**						
**<18.5**	4,298 (6.6%)	44 (5.4%)	159 (1.1%)	37 (5.5%)	53 (1.0%)	9 (3.2%)
**[18.5,25)**	33,912 (51.7%)	391 (47.9%)	7,878 (53.1%)	323 (47.8%)	2,700 (49.9%)	129 (46.1%)
**[25,30)**	19,449 (29.6%)	275 (33.7%)	4,781 (32.3%)	228 (33.7%)	1,865 (34.4%)	96 (34.3%)
**≥30**	7,940 (12.1%)	106 (13.0%)	2,008 (13.5%)	88 (13.0%)	795 (14.7%)	46 (16.4%)
**Height* (cm), *N* (%)**						
**<152.91**	625 (1.0%)	4 (0.5%)	144 (1.0%)	4 (0.6%)	55 (1.0%)	2 (0.7%)
**[152.91, 159.65)**	6,240 (9.5%)	67 (8.2%)	1,528 (10.3%)	53 (7.8%)	591 (10.9%)	27 (9.6%)
**[159.65, 165.96)**	20,366 (31.0%)	237 (29.0%)	5,014 (33.8%)	195 (28.9%)	1,870 (34.6%)	88 (31.4%)
**[165.96, 172.70)**	24,645 (37.6%)	333 (40.8%)	5,847 (39.4%)	280 (41.4%)	2,108 (38.9%)	111 (39.6%)
**≥172.70**	10,053 (15.3%)	135 (16.5%)	2,284 (15.4%)	111 (16.4%)	787 (14.5%)	43 (15.4%)
**Missing**	3,670 (5.6%)	40 (4.9%)	9 (0.1%)	33 (4.9%)	2 (0.0%)	9 (3.2%)
**Alcohol consumption* (g/day), *N* (%)**						
**<5**	11,738 (17.9%)	130 (15.9%)	2,830 (19.1%)	108 (16.0%)	1,050 (19.4%)	41 (14.6%)
**[5,15)**	40,501 (61.7%)	506 (62.0%)	9,670 (65.2%)	423 (62.6%)	3,484 (64.4%)	184 (65.7%)
**[15,25)**	5,918 (9.0%)	93 (11.4%)	1,478 (10.0%)	76 (11.2%)	599 (11.1%)	32 (11.4%)
**[25,35)**	1,213 (1.9%)	13 (1.6%)	290 (2.0%)	8 (1.2%)	116 (2.1%)	3 (1.1%)
**[35,45)**	1,195 (1.8%)	19 (2.3%)	274 (1.8%)	16 (2.4%)	103 (1.9%)	6 (2.1%)
**≥45**	219 (0.3%)	3 (0.4%)	57 (0.4%)	2 (0.3%)	22 (0.4%)	1 (0.4%)
**Missing**	4,815 (7.3%)	52 (6.4%)	227 (1.5%)	43 (6.4%)	39 (0.7%)	13 (4.6%)
**BI-RADS, *N* (%)**						
**A**	8,209 (12.5%)	64 (7.8%)	2,173 (14.7%)	52 (7.7%)	949 (17.5%)	28 (10.0%)
**B**	24,551 (37.4%)	334 (40.9%)	5,981 (40.3%)	279 (41.3%)	2,443 (45.1%)	113 (40.4%)
**C**	25,982 (39.6%)	329 (40.3%)	5,424 (36.6%)	272 (40.2%)	1,704 (31.5%)	116 (41.4%)
**D**	6,857 (10.5%)	89 (10.9%)	1,248 (8.4%)	73 (10.8%)	317 (5.9%)	23 (8.2%)
**Number of 1st degree relatives with BC, *N* (%)**						

Yang X, *et al. J Med Genet* 2022; 59:1196–1205. doi: 10.1136/jmg-2022-108806

**0**	58,102 (88.6%)	660 (80.9%)	13,050 (88.0%)	547 (80.9%)	4,783 (88.4%)	225 (80.4%)
**1**	7,183 (10.9%)	145 (17.8%)	1,699 (11.5%)	124 (18.3%)	602 (11.1%)	53 (18.9%)
**≥2**	314 (0.5%)	11 (1.4%)	77 (0.5%)	5 (0.7%)	28 (0.5%)	2 (0.7%)
**Standardized PRS, mean (sd)**						
	-	-	-0.0089 (1.03)	0.44 (1.01)	-0.068 (1.03)	0.51 (1.01)
***BRCA1*, *N* (%)**						
**Negative**	-	-	-	-	5,410 (99.9%)	279 (99.6%)
**Positive**	-	-	-	-	3 (0.1%)	1 (0.4%)
**Untested**	-	-	-	-	0 (0.0%)	0 (0.0%)
***BRCA2*, *N* (%)**						
**Negative**	-	-	-	-	5,403 (99.8%)	278 (99.3%)
**Positive**	-	-	-	-	10 (0.2%)	2 (0.7%)
**Untested**	-	-	-	-	0 (0%)	0 (0%)
***PALB2*, *N* (%)**						
**Negative**	-	-	-	-	5,408 (99.9%)	278 (99.3%)
**Positive**	-	-	-	-	5 (0.1%)	2 (0.7%)
**Untested**	-	-	-	-	0 (0.0%)	0 (0.0%)
***ATM*, *N* (%)**						
**Negative**	-	-	-	-	5,402 (99.8%)	278 (99.3%)
**Positive**	-	-	-	-	11 (0.2%)	2 (0.7%)
**Untested**	-	-	-	-	0 (0.0%)	0 (0.0%)
***CHEK2*, *N* (%)**						
**Negative**	-	-	-	-	5,356 (99.0%)	275 (98.2%)
**Positive**	-	-	-	-	57 (1.0%)	5 (1.8%)
**Untested**	-	-	-	-	0 (0%)	0 (0%)
***RAD51C*, *N* (%)**						
**Negative**	-	-	-	-	5,408 (99.9%)	280 (100.0%)
**Positive**	-	-	-	-	5 (0.1%)	0 (0.0%)
**Untested**	-	-	-	-	0 (0.0%)	0 (0.0%)
***RAD51D*, *N* (%)**						

	**Negative**	-	-	-	-	5,413 (100.0%)	279 (99.6%)
	**Positive**	-	-	-	-	0(0.0%)	1 (0.4%)
	**Untested**	-	-	-	-	0 (0.0%)	0 (0.0%)
***BARD1*, *N* (%)**							
	**Negative**	-	-	-	-	5,356 (99.9%)	280 (100.0%)
	**Positive**	-	-	-	-	6 (0.1%)	0 (0.0%)
	**Untested**	-	-	-	-	0 (0.0%)	0 (0.0%)

* These are questionnaire based risk factors (QRFss), considered in BOADICEA. Risk factor categories as defined in the BOADICEA model.

- Not applicable

Table s3 Tumour characteristics for incident breast cancer patients.

	Full cohort (*N*=816)	Sub cohort with PRS (*N*=676)	Sub cohort with PRS and PV (*N*=280)
**Stage, *N* (%)**			
**0**	4 (0.5%)	4 (0.6%)	3 (1.1%)
**1**	444 (54.4%)	357 (52.8%)	146 (52.1%)
**2**	216 (26.5%)	182 (26.9%)	69 (24.6%)
**3**	74 (9.1%)	65 (9.6%)	31 (11.1%)
**4**	6 (0.7%)	5 (0.7%)	1 (0.4%)
**missing**	72 (8.8%)	63 (9.3%)	30 (10.7%)
**Morphology, *N* (%)**			
**ductal**	622 (76.2%)	516 (76.3%)	218 (77.9%)
**lobular**	97 (11.9%)	83 (12.3%)	39 (13.9%)
**Medullary**	2 (0.2%)	1 (0.1%)	0 (0.0%)
**mixed (ductal and lobular)**	13 (1.6%)	11 (1.6%)	3 (1.1%)
**missing**	82 (10.0%)	65 (9.6%)	20 (7.1%)
**ER status, *N* (%)**			
**negative**	97 (11.9%)	80 (11.8%)	31 (11.1%)
**positive**	671 (82.2%)	558 (82.5%)	232 (82.9%)
**missing**	48 (5.9%)	38 (5.6%)	17 (6.1%)

Table s4 Reclassification table comparing the model considering family history (FH), questionnaire-based risk factors (QRFs), mammographic density (MD) in BI-RADS and polygenic risk score (PRS) and the model considering FH using the subcohort samples with PRS. The table shows the weighted number of women in each category of risk (under each model). The weighted number of incident breast cancer patients is shown in the brackets.

**Model: FH**	**Model: FH+QRFs+MD+PRS**		
	Five-year risk < 3%	Five-year risk ≥ 3%	Total
**All women**			
**Five-year risk < 3%**	64049<br>(733)	1894<br>(68)	65943<br>(801)
**Five-year risk ≥ 3%**	332<br>(7)	189<br>(7)	521<br>(14)
**Total**	64381<br>(740)	2083<br>(75)	66464<br>(815)
**Premenopausal women**			
**Five-year risk < 3%**	30027<br>(239)	722<br>(23)	30749<br>(262)
**Five-year risk ≥ 3%**	9<br>(0)	30<br>(1)	39<br>(1)
**Total**	30036<br>(239)	752<br>(24)	30788<br>(263)
**Postmenopausal women**			
**Five-year risk < 3%**	34022<br>(494)	1172<br>(45)	35194<br>(539)
**Five-year risk ≥ 3%**	323<br>(7)	159<br>(6)	482<br>(13)
**Total**	34345<br>(501)	1331<br>(51)	35676<br>(552)

Yang X, *et al. J Med Genet* 2022; 59:1196–1205. doi: 10.1136/jmg-2022-108806

Table s5 Sensitivity and specificity at different risk thresholds and proportions of ER-negative and ER-positive cancers detected in each risk category.

Risk threshold/ category	Model: FH+QRFs+MD+PRS†				Model: FH+QRFs+MD+PRS+PV‡			
	BCs=676		BCs with ER status=644		BCs=280		BCs with ER status=267	
	Sensitivity	Specificity	ER-negative cancers (%*)	ER-positive cancers (%**)	Sensitivity	Specificity	ER-negative cancers (%*)	ER-positive cancers (%**)
**<1.10%**	-	-	34 (42.5%)	166 (29.4%)	-	-	15 (48.4%)	69 (29.2%)
**[1.10#-1.67%)**	0.69 (0.66, 0.72)	0.59 (0.59, 0.59)	22 (27.5%)	181 (32.1%)	0.66 (0.62, 0.69)	0.63 (0.63, 0.63)	5 (16.1%)	72 (30.5%)
**[1.67#-3.00%)**	0.38 (0.34, 0.41)	0.81 (0.81, 0.82)	22 (27.5%)	161 (28.5%)	0.34 (0.31, 0.37)	0.83 (0.83, 0.84)	8 (25.8%)	70 (29.7%)
**≥3%#**	0.09 (0.01, 0.11)	0.97 (0.97, 0.97)	2 (2.5%)	56 (9.9%)	0.09 (0.07, 0.11)	0.97 (0.96, 0.97)	3 (9.7%)	25 (10.6%)

#Risk thresholds (≥) for calculating sensitivity/specificity

*among ER-negative; **among ER-positive

†Using the weighted sub-cohort with PRS (*N*=15,502); ‡Using the weighted sub-cohort with PRS and PV (*N*=5,693). Therefore, the results were not directly comparable due to the different sample sizes.

FH: family history; QRFs: questionnaire-based risk factors; MD: mammographic density in BI-RADS; PRS: polygenic risk score; PV: pathogenic variants in *BRCA1*, *BRCA2*, *PALB2*, *CHECK2*, *ATM*, *RAD51C*, *RAD51D*, and *BARD1*.

Yang X, *et al. J Med Genet* 2022; 59:1196–1205. doi: 10.1136/jmg-2022-108806

Table s6 Sensitivity and specificity at different percentiles of the predicted risk distribution, under the model considering family history (FH), questionnaire-based risk factors (QRFs), mammographic density in BI-RADS (MD) and polygenic risk score (PRS).

Risk distribution threshold	Equivalen 5-year risk threshold	sensitivity	specificity
**Top 2.5%**	3.26%	0.07 (0.05, 0.09)	0.98 (0.98, 0.98)
**Top 5%**	2.72%	0.13 (0.11, 0.15)	0.96 (0.95, 0.96)
**Top 10%**	2.20%	0.23 (0.20, 0.26)	0.91 (0.91, 0.91)
**Top 20%**	1.69%	0.37 (0.34, 0.41)	0.82 (0.82, 0.82)
**Top 30%**	1.39%	0.53 (0.49, 0.56)	0.73 (0.72, 0.73)
**Top 40%**	1.18%	0.65 (0.61, 0.68)	0.63 (0.63, 0.63)
**Top 50%**	1.00%	0.75 (0.72, 0.78)	0.53 (0.53, 0.54)

Yang X, *et al. J Med Genet* 2022; 59:1196–1205. doi: 10.1136/jmg-2022-108806

Table s7 Reclassification table comparing the model that considers family history (FH), questionnaire-based risk factors (QRFs), mammographic density (MD) in BI-RADS, polygenic risk score (PRS) and pathogenic variants (PV) in the five major breast cancer (BC) susceptibility genes and the model considering FH, QRFs, MD and PRS, based on the subcohort samples with PRS and PV status. The table shows the weighted number of women in each category of risk. The weighted number of incident BC patients is shown in the brackets.

**Model: FH+QRFs+MD+PRS**	**Model: FH+QRFs+MD+PRS+PV**		
	Five-year risk < 3%	Five-year risk ≥ 3%	Total
**Five-year risk < 3%**	63412<br>(736)	647<br>(23)	64059<br>(759)
**Five-year risk ≥ 3%**	228<br>(7)	1702<br>(49)	1930<br>(59)
**Total**	63640<br>(743)	2349<br>(67)	65989<br>(816)

Table s8 Risk reclassification of the 110 pathogenic variant (PV) carriers using five-year predicted breast cancer risk under different models. FH: family history; QRFs: questionnaire-based risk factors; MD: mammographic density in BI-RADS; PRS: polygenic risk score; PV: pathogenic variants in *BRCA1*, *BRCA2*, *PALB2*, *CHECK2*, *ATM*, *RAD51C*, *RAD51D*, and *BARD1*.

**Model**	**Risk <1.67%**	**Risk: 1.67-3%**	**Risk: ≥3%**	**E/O**
FH Only	45%	53%	2%	0.35
+ PRS+ MD+ QRFs	68%	27%	5%	0.31
+ PRS+ MD+ QRFs+ PV	25%	23%	52%	0.99
PV only	3%	36%	61%	0.99
+ PRS+MD+QRFs+FH	25%	23%	52%	0.99

Yang X, *et al. J Med Genet* 2022; 59:1196–1205. doi: 10.1136/jmg-2022-108806

Table s9 Calibration and discrimination of five-year predicted breast cancer risks in women 40 years old or older using different risk factor combinations.

Model	AUC (95%CI)	Harrell's C (95%CI)	E/O (95%CI)	Calibration slope (95%CI)
**Entire cohort with information on FH, QRF and MD (*N*=66,152; *N*.BCs=816)**				
**FH**	0.62 (0.60, 0.64)	0.62 (0.61, 0.65)	1.12 (1.05, 1.20)	1.03 (1.01, 1.05)
**QRF**	0.62 (0.60, 0.64)	0.62 (0.60, 0.64)	0.95 (0.89, 1.02)	0.99 (0.97, 1.01)
**MD**	0.62 (0.60, 0.64)	0.62 (0.60, 0.64)	0.94 (0.88, 1.01)	0.99 (0.97, 1.01)
**FH+QRF**	0.63 (0.61, 0.65)	0.63 (0.61, 0.65)	1.02 (0.95, 1.09)	1.00 (0.99, 1.02)
**FH+QRF+MD**	0.64 (0.62, 0.66)	0.63 (0.62, 0.66)	0.92 (0.86, 0.99)	0.98 (0.96, 1.00)
**Sub-cohort with information on FH, QRF, MD and PRS (*N*=15,427; *N*.BCs=676)**				
**FH**	0.61 (0.59, 0.63)	0.62 (0.60, 0.64)	1.12 (1.04, 1.21)	1.03 (1.01, 1.05)
**QRF**	0.62 (0.60, 0.65)	0.63 (0.61, 0.65)	0.94 (0.87, 1.01)	0.99 (0.97, 1.00)
**MD**	0.62 (0.60, 0.64)	0.64 (0.61, 0.66)	0.93 (0.86, 1.01)	0.98 (0.97, 1.00)
**PRS**	0.66 (0.64, 0.68)	0.67 (0.65, 0.69)	1.06 (0.98, 1.15)	1.01 (1.00, 1.03)
**FH+QRF+MD**	0.65 (0.63, 0.67)	0.65 (0.63, 0.67)	0.90 (0.84, 0.97)	0.98 (0.96, 1.00)
**FH+QRF+PRS**	0.68 (0.66, 0.70)	0.68 (0.66, 0.70)	1.01 (0.93, 1.09)	1.00 (0.98, 1.02)
**FH+QRF+PRS+MD**	0.69 (0.67, 0.71)	0.68 (0.67, 0.70)	0.91 (0.85, 0.98)	0.98 (0.96, 1.00)
**Sub-cohort with information on FH, QRF, MD, PRS and PV status (*N*=5,653; *N*.BCs=280)**				
**FH+QRF+PRS+MD**	0.68 (0.64, 0.72)	0.7 (0.66, 0.72)	0.88 (0.74, 1.04)	0.97 (0.95, 0.99)
**FH+QRF+PRS+MD+PV**	0.69 (0.66, 0.73)	0.7 (0.67, 0.73)	0.88 (0.75, 1.04)	0.97 (0.95, 0.99)

BC: breast cancer; E: expected number of breast cancers in the five-year period; O: observed number breast cancers; FH: family history; QRFs: questionnaire-based risk factors; MD: mammographic density in BI-RADS; PRS: polygenic risk score; PV: pathogenic variants in *BRCA1*, *BRCA2*, *PALB2*, *CHECK2*, *ATM*, *RAD51C*, *RAD51D*, and *BARD1*.

Yang X, *et al. J Med Genet* 2022; 59:1196–1205. doi: 10.1136/jmg-2022-108806

Table s10 Calibration and discrimination of five-year predicted breast cancer risks by menopausal status. Analyses restricted to women 40 years old or older.

Menopausal status	*N*. unaffected	*N*. BCs	Model	AUC (95%CI)	Harrell's C (95%CI)	E/O (95%CI)	Calibration slope (95%CI)
**Sub-cohort with information on FH, QRFs, MD, and PRS (*N*=15,427; *N*.BCs=676)**							
**Pre-menopausal**	5,427	212	FH+QRFs+PRS	0.67 (0.63, 0.71)	0.66 (0.62, 0.7)	1.09 (0.95, 1.25)	1.02 (0.99, 1.05)
			FH+QRFs+PRS+MD	0.68 (0.65, 0.72)	0.68 (0.65, 0.71)	1.13 (0.99, 1.3)	1.03 (0.99, 1.06)
**Post-menopausal**	9,324	464	FH+QRFs+PRS	0.66 (0.63, 0.68)	0.65 (0.63, 0.68)	0.97 (0.89, 1.06)	0.99 (0.97, 1.02)
			FH+QRFs+PRS+MD	0.67 (0.64, 0.69)	0.67 (0.64, 0.68)	0.81 (0.74, 0.89)	0.95 (0.93, 0.97)
**Sub-cohort with information on FH, QRFs, MD, PRS, and PV status (*N*=5,653; *N*.BCs=280)**							
**Pre-menopausal**	1,176	77	FH+QRFs+PRS+PV	0.69 (0.6, 0.78)	0.67 (0.59, 0.73)	1.1 (0.77, 1.58)	1.02 (0.99, 1.05)
			FH+QRFs+PRS+PV+MD	0.68 (0.6, 0.75)	0.67 (0.59, 0.74)	1.11 (0.78, 1.6)	1.02 (0.98, 1.05)
**Post-menopausal**	4,197	203	FH+QRFs+PRS+PV	0.68 (0.63, 0.73)	0.69 (0.65, 0.72)	0.95 (0.79, 1.13)	0.99 (0.96, 1.01)
			FH+QRFs+PRS+PV+MD	0.68 (0.64, 0.73)	0.69 (0.66, 0.73)	0.78 (0.66, 0.94)	0.94 (0.92, 0.96)

BC: Breast Cancer; FH: family history; QRFs: questionnaire-based risk factors; MD: mammographic density in BI-RADS; PRS: polygenic risk score; PV: pathogenic variants in *BRCA1*, *BRCA2*, *PALB2*, *CHECK2*, *ATM*, *RAD51C*, *RAD51D*, and *BARD1*.

Yang X, *et al. J Med Genet* 2022; 59:1196–1205. doi: 10.1136/jmg-2022-108806

Figure s1 Predicted five-year risk distribution for the incident breast cancer patients (affected) and healthy women (unaffected) under different models using the entire cohort (*N*=66,415). The box represents the interquartile range (IQR) and the central line within the box represents the mean. The x-axis is on the log-scale.

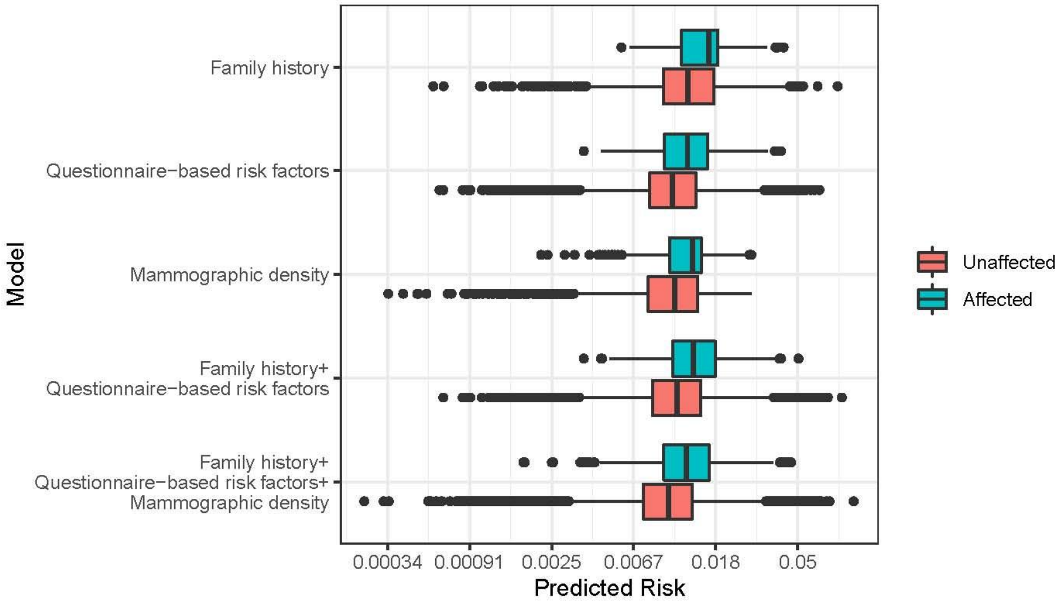

Yang X, *et al. J Med Genet* 2022; 59:1196–1205. doi: 10.1136/jmg-2022-108806

Figure s2 Observed and predicted five-year breast cancer risks in deciles of predicted risks using the entire cohort under different models Women were grouped into deciles of predicted risks. Each dot represents the mean observed and predicted risk in the decile and the vertical segments represent 95% confidence intervals. The dashed line is the diagonal line with slope equal to 1 (corresponding to E/O ratio of 1 for each decile). When the confidence interval crosses the diagonal, the decile-predicted risk is not significantly different from the observed risk. When a dot and the associated confidence interval fall above the diagonal, there is a suggestion for underprediction of risk; when a dot and associated confidence interval fall below the diagonal there is a suggestion for overprediction of risk.

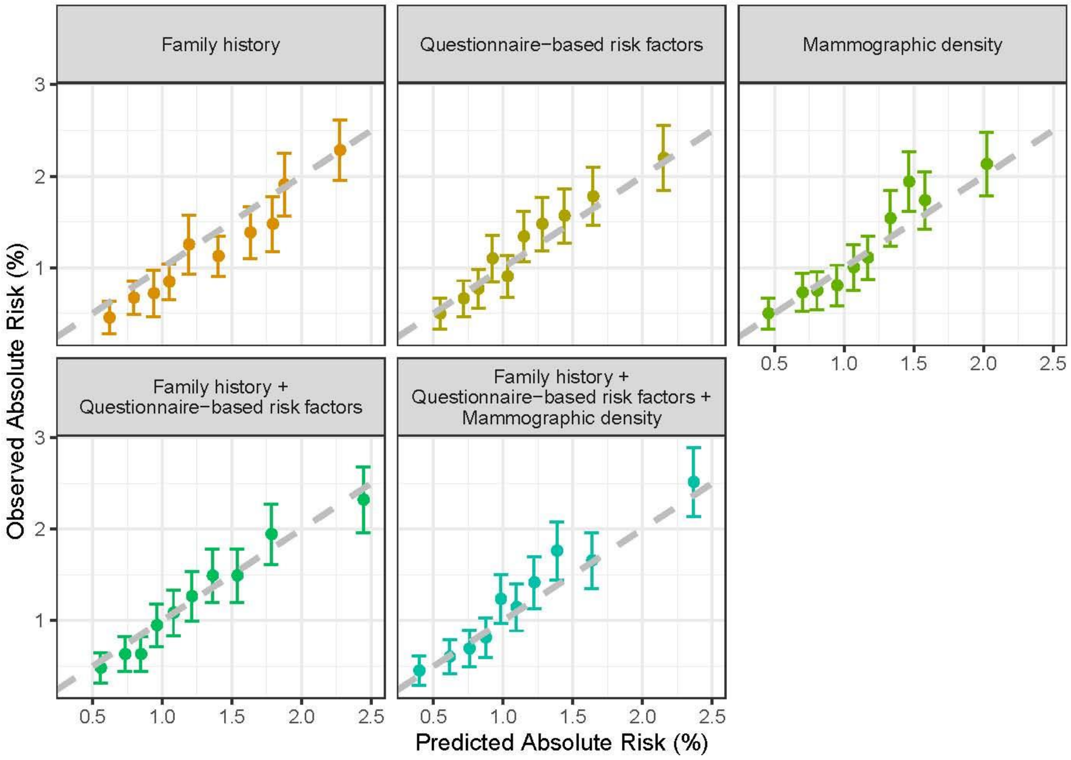

Figure s3 Predicted five-year risk distribution for the incident breast cancer patients (affected) and healthy women (unaffected) under different models using the subcohort samples with polygenic risk score (PRS) information(*N*=15,502). The box represents the interquartile range (IQR) and the central line within the box represents the mean. . The x-axis is on the log-scale.

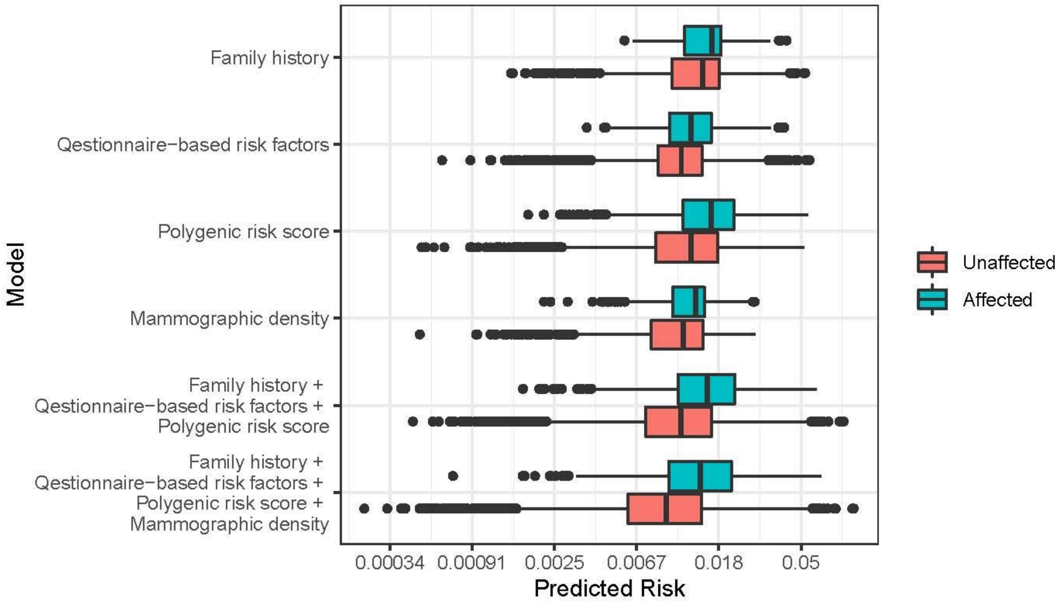

Yang X, *et al. J Med Genet* 2022; 59:1196–1205. doi: 10.1136/jmg-2022-108806

Figure s4 Predicted five-year risk distribution for the incident breast cancer patients (affected) and healthy women (unaffected) under different models using the subcohort samples with polygenic risk score (PRS) and pathogenic variants (PV) information(*N*=5,693). The box represents the interquartile range (IQR) and the central line within the box represents the mean. The x-axis is on the log-scale.

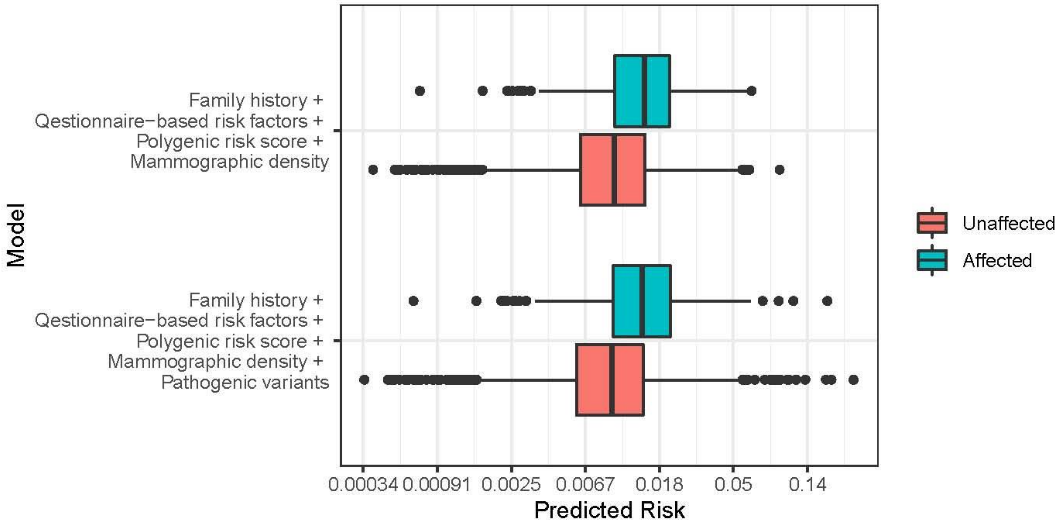

Yang X, *et al. J Med Genet* 2022; 59:1196–1205. doi: 10.1136/jmg-2022-108806

Figure s5 Predicted five-year risk distributions for carriers and non-carriers of pathogenic variants (PV) in the eight major breast cancer (BC) susceptibility genes: *BRCA1*, *BRCA2*, *PALB2*, *CHECK2*, *ATM*, *RAD51C*, *RAD51D* and *BARD1* under the models considering family history, questionnaire-based risk factor, polygenic risk score, mammographic density in BI-RADS and pathogenic variant status using the subcohort samples with PRS and PV information (*N*=5,693). The box represents the interquartile range (IQR) and the central line within the box represents the mean. . The x-axis is on the log-scale.

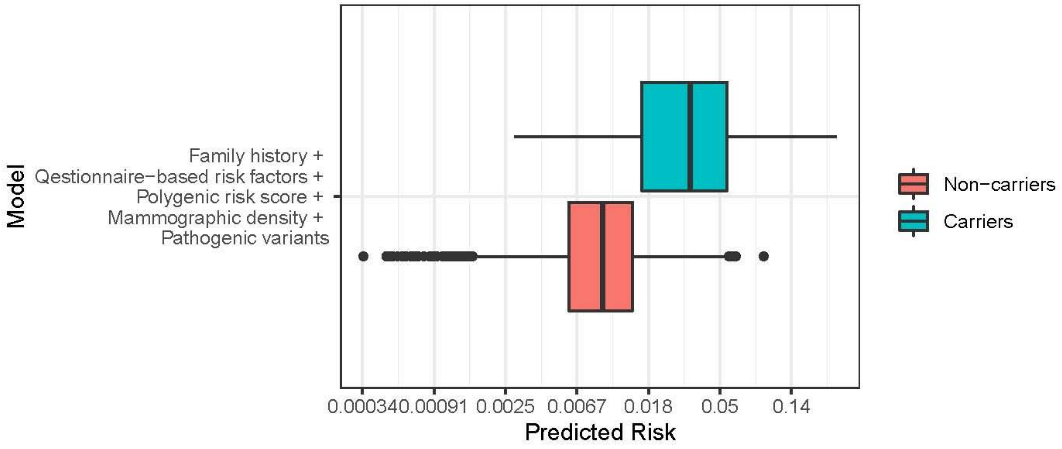

Yang X, *et al. J Med Genet* 2022; 59:1196–1205. doi: 10.1136/jmg-2022-108806

Figure s6 The distribution of BIRADS categories across deciles of predicted risks in postmenopausal women in the subcohort samples with polygenic risk score (PRS) information (*N*=9,788). Each bar represents the proportion of women with BIRADS A, B, C, or D among total number of unaffected or affected women. Affected: incident breast cancer patients during the five-year risk prediction horizon; Unaffected: women without a breast cancer diagnosis during the five-year risk prediction horizon.

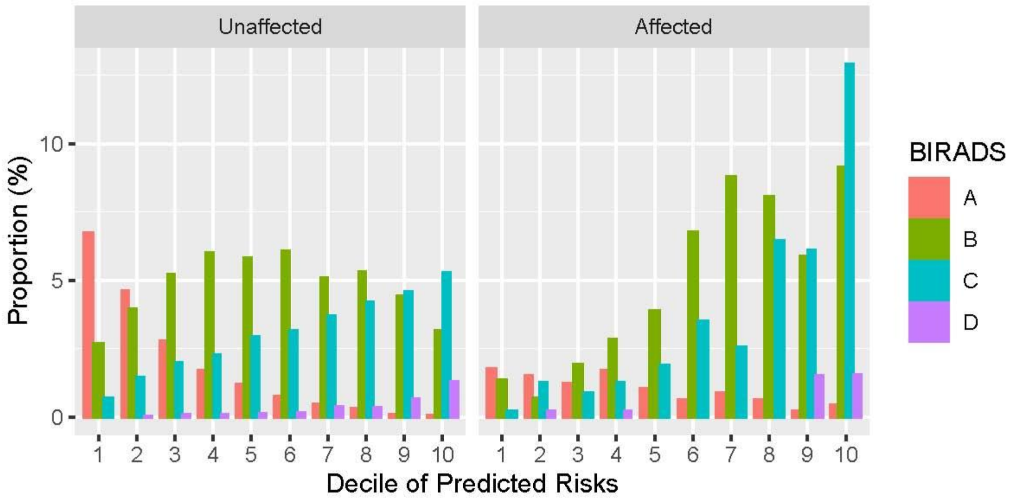

Figure s7. Observed and expected five-year breast cancer risks in deciles of predicted risks in women 40 years old or older. The sub-cohort of samples with PRS information (*N*=15,427) was used here under the model considering considering family history, questionnaire-based risk factors, polygenic risk score and mammographic density in BI-RADS. Women were grouped into deciles of predicted risks.

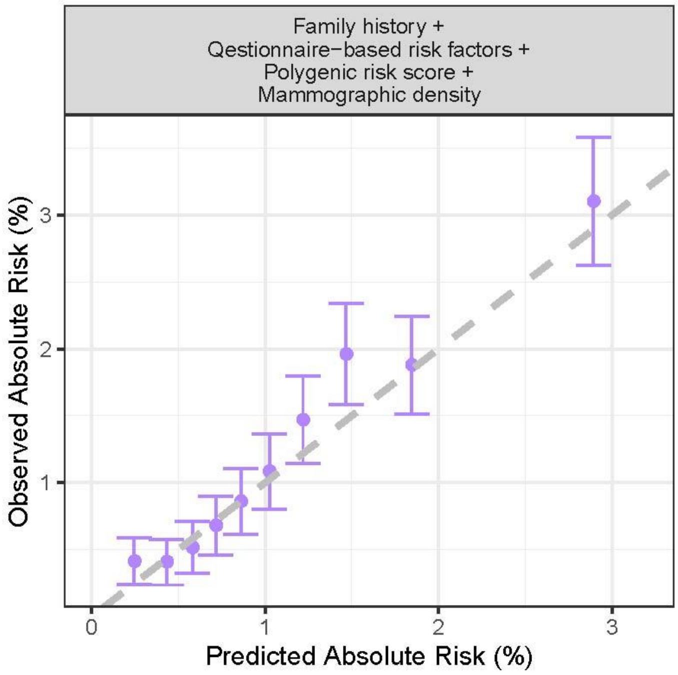

Yang X, *et al. J Med Genet* 2022; 59:1196–1205. doi: 10.1136/jmg-2022-108806

Figure s8. Observed and expected five-year breast cancer risks in deciles of predicted risks in women 40 years old or older. The sub-cohort of samples with PRS and PV status information (*N*=5,653) was used here underthe model considering all risk factors including family history, questionnaire-based risk factors, polygenic risk score, mammographic density in BI-RADS and pathogenic variant status in the eight major breast cancer (BC) susceptibility genes: *BRCA1*, *BRCA2*, *PALB2*, *CHECK2*, *ATM*, *RAD51C*, *RAD51D* and *BARD1*. Women were grouped into deciles of predicted risks.

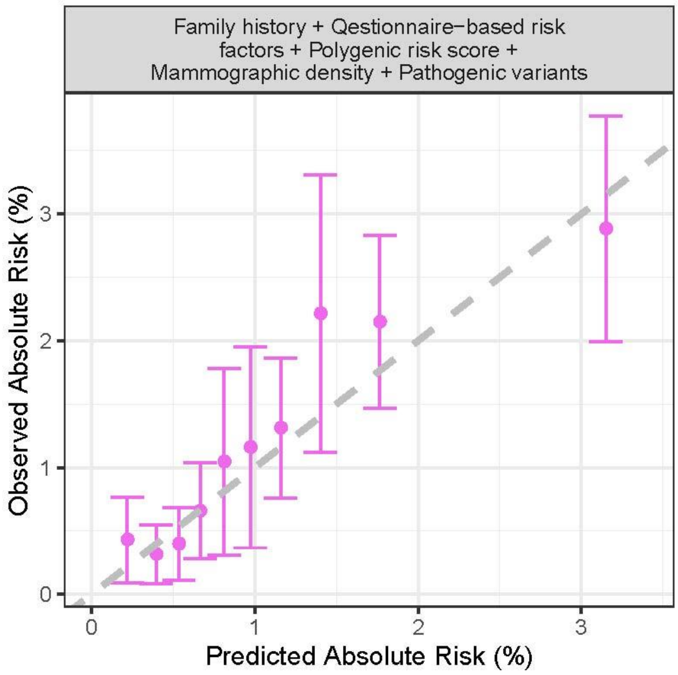

Yang X, *et al. J Med Genet* 2022; 59:1196–1205. doi: 10.1136/jmg-2022-108806
